# Periodate-treated, non-anticoagulant heparin-carrying polystyrene (NAC-HCPS) affects angiogenesis and inhibits subcutaneous induced tumour growth and metastasis to the lung

**DOI:** 10.1038/sj.bjc.6600307

**Published:** 2002-06-05

**Authors:** K Ono, M Ishihara, K Ishikawa, Y Ozeki, H Deguchi, M Sato, H Hashimoto, Y Saito, H Yura, A Kurita, T Maehara

**Affiliations:** Department of Surgery II, National Defense Medical College, 3-2, Namiki, Tokorozawa, Saitama, 359-8513 Japan; Research Institute, Division of Biomedical Engineering, National Defense Medical College, 3-2, Namiki, Tokorozawa, Saitama, 359-8513 Japan; Department of Surgery I, National Defense Medical College, 3-2, Namiki, Tokorozawa, Saitama, 359-8513 Japan; NeTech Inc., KSP East Wing 502, Sakado 3-2-1, Takatsu, Kawasaki, Kanagawa 213-0012, Japan

**Keywords:** periodate-treated, non-anticoagulant heparin-carrying polystyrene (NAC-HCPS), heparin, angiogenesis, metastasis, tumour growth

## Abstract

Periodate-treated, non-anticoagulant heparin-carrying polystyrene consists of about ten periodate-oxidized, alkaline-degraded low molecular weight-heparin chains linked to a polystyrene core and has a markedly lower anti-coagulant activity than heparin. In this study, we evaluated the effect of non-anticoagulant heparin-carrying polystyrene on tumour growth and metastasis. Non-anticoagulant heparin-carrying polystyrene has a higher activity to inhibit vascular endothelial growth factor-165-, fibroblast growth factor-2- or hepatocyte growth factor-induced human microvascular endothelial cell growth than heparin, ten periodate-oxidized-heparin and ten periodate-oxidized-low molecular weight-heparin, which is probably due to the heparin-clustering effect of non-anticoagulant heparin-carrying polystyrene. Non-anticoagulant heparin-carrying polystyrene inhibited human microvascular endothelial cell, B16 melanoma and Lewis lung cancer cell adhesion to Matrigel-coated plates. Non-anticoagulant heparin-carrying polystyrene also showed strong inhibitory activities in the tubular formation of endothelial cells on Matrigel and B16-melanoma and Lewis lung cancer cell invasion in a Matrigel-coated chamber assay. *In vivo* studies showed that growth of subcutaneous induced tumours and lung metastasis of B16-melanoma and Lewis lung cancer cells were more effectively inhibited by non-anticoagulant heparin-carrying polystyrene than ten periodate-oxidized-heparin and ten periodate-oxidized-low molecular weight-heparin. Furthermore, non-anticoagulant heparin-carrying polystyrene markedly reduced the number of CD34-positive vessels in subcutaneous Lewis lung cancer tumours, indicating a strong inhibition of angiogenesis. These results suggest that non-anticoagulant heparin-carrying polystyrene has an inhibitory activity on angiogenesis and tumour invasion and may be very useful in cancer therapy.

*British Journal of Cancer* (2002) **86**, 1803–1812. doi:10.1038/sj.bjc.6600307 www.bjcancer.com

© 2002 Cancer Research UK

## 

Heparin (Hep)/heparan sulphate (HS) are members of the glycosaminoglycans (GAGs) and are normally present as proteoglycans (PGs), in which a number of Hep/HS-chains are covalently attached to a core protein. While HS is widely distributed on cell surfaces and in extracellular matrices in most animal tissues, Hep is synthesised by mast cells in connective tissue and stored in cytoplasmic granules (Ishihara and Ono, 1998). Heparin is isolated on a commercial basis from animal tissue (pig or bovine intestinal mucosa, or bovine lung etc.) and has been extensively used as an anti-thrombotic drug for a long time (Ishihara and Ono, 1998). The biological role of Hep/HS is highly diverse. Aside from its well-known anti-coagulant action, the molecules are found to be associated with growth factors and cytokines in various biological processes, as well as being involved in cell adhesion, recognition, migration, and regulation of various enzymatic activities (Lindahl *et al*, 1994; Kjellen and Lindahl, 1991).

Several studies have reported both inhibitory and stimulatory effects of Hep on tumour growth and metastasis (Zacharski and Ornstein, 1998; Engelberg, 1999; Smorenburg and Van Noorden, 2001). Besides the anticoagulant function, Hep binds to various growth factors, cytokines, and extracellular matrix (ECM) proteins and consequently is able to affect proliferation and migration of cancer cells and angiogenesis in tumours (Lindahl *et al*, 1994). Furthermore, Heps have been found to inhibit expression of oncogenes and to affect the immune system (Smorenburg and Van Noorden, 2001). Heparins also show both inhibitory and stimulatory effects of various proteolytic enzymes, which are essential for invasion of cancer cells and angiogenesis through the ECM (Zacharski and Ornstein, 1998; Engelberg, 1999; Smorenburg and Van Noorden, 2001). Due to the wide variety of activities of Heps, the ultimate effect of a Hep treatment on cancer progression is unpredictable. In addition, the use of a high-dose Hep has been limited by its strong anti-coagulant property, which may cause severe bleeding complications (Levine *et al*, 1989; Lapierre *et al*, 1996).

Periodate-treated, non-anticoagulant Hep-carrying polystyrene (NAC-HCPS) has been described previously as a synthetic glyco-conjugate that is soluble in water and has an amphiphilic structural unit consisting of hydrophilic polysaccharides and hydrophobic polystyrene moieties (Ishihara *et al*, 2000a). It has been estimated that the molecular size of NAC-HCPS is approximately 80–120 kDa and comprises of over ten periodate-oxidised, alkaline-degraded low molecular weight (IO_4_-LMW-) Hep chains enriched in trisulphated disaccharide structures linked to its polystyrene core (Ishihara *et al*, 2000a,b). Non-anticoagulant Hep-carrying polystyrene shows a significantly reduced anticoagulant activity and enhanced abilities to interact with various heparin-binding growth factors, such as fibroblast growth factor-2 (FGF-2), vascular endothelial growth factor-165 (VEGF_165_) and hepatocyte growth factor (HGF), which are known to stimulate angiogenesis (Ishihara *et al*, 2000b).

The present study evaluates the effect of NAC-HCPS on malignant processes *in vitro* and *in vivo*, thereby focusing on angiogenesis. Non-anticoagulant Hep-carrying polystyrene inhibit, (i) heparin-binding growth factor-induced human microvascular endothelial cell (HMVEC) proliferation, (ii) HMVEC adhesion onto Matrigel, (iii) formation of capillary-like tubular structures on Matrigel by HMVECs *in vitro*, and (iv) vascularisation in tumour tissue *in vivo*. In addition, NAC-HCPS is an inhibitor for experimental subcutaneous tumour growth and metastasis to the lung using B16 melanoma (B16) and Lewis lung cancer (3LL) cells.

## MATERIALS AND METHODS

### Preparation of modified Heps and NAC-HCPS

**Figure 1:**
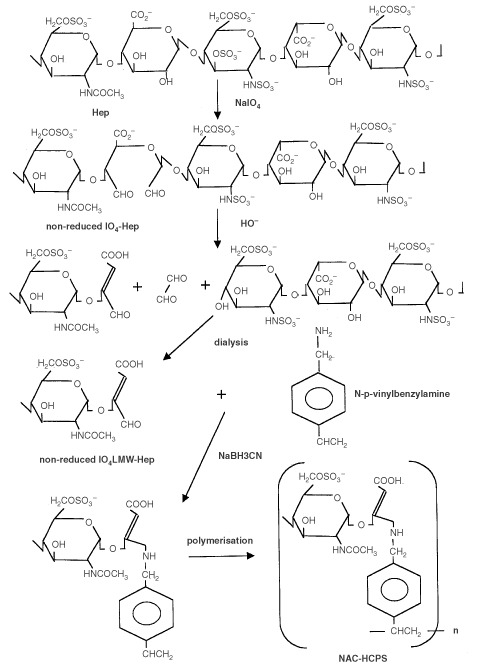
Reaction scheme for the preparation of IO_4_-Hep, IO_4_-LMW-Hep and NAC-HCPS.

Non-anticoagulant Hep-carrying polystyrene was prepared as has been reported previously (Ishihara *et al*, 2000a). An outline of the used chemical reaction route to synthesise modified heparins and NAC-HCPS is presented in Figure 1

. Briefly, 25 g of Hep from porcine intestine (185.8 USP Units mg^−1^) dissolved in 400 ml of 0.1 M NaIO_4_ in 0.05 M sodium acetate buffer (pH 5) was stirred at 4°C for 3 days. The unreacted NaIO_4_ was then neutralised by addition of glycerol (25 ml), and the reaction mixture was subsequently dialysed and lyophilised. The product (non-reduced periodate-oxidised heparin; non-reduced IO_4_-Hep) was then degraded in an alkaline solution (pH 12) at room temperature for 30 min, and the degraded product was recovered after dialysis and lyophilisation as non-reduced periodate-oxidised, alkaline-degraded (non-reduced IO_4_-LMW-) Hep. To prepare reduced IO_4_-Hep and reduced IO_4_-LMW-Hep as control compounds in order to compare with NAC-HCPS, both non-reduced IO_4_-Hep and non-reduced IO_4_-LMW-Hep were reduced by mixing them with 0.2 M sodium borohydride in 0.25 M sodium bicarbonate for 3 h at 4°C. The excess borohydride in both reactions was destroyed by adding acetic acid (pH 5). The reduced IO_4_-Hep and reduced IO_4_-LMW-Hep were then recovered after neutralising with NaOH, dialysis and lyophilisation.

The non-reduced IO_4_-LMW-Hep (500 mg) and *p*-styrenemethylamine (250 mg) were dissolved in 20 ml of 50 mM *N,N,N*′*,N*′-tetramethyl-ethylenediamine (pH 4.75), after which 1 ml of 0.8 mM NaCNBH_3_ was added. The reaction mixture was stirred for 24 h at room temperature, dialysed and lyophilised to yield a white powder (heparin-styrene monomer). This powder (100 mg) and 2 mg of potassium peroxodisulphate were dissolved in 1 ml of distilled water and the polymerisation was carried out at 60°C for 24 h under dried N_2_ gas. The reaction solution was then slowly poured into an excess amount of ethanol to yield a polymeric precipitate. Water-soluble impurities were separated from the precipitate using ultra-filtration and finally the NAC-HCPS was obtained as a white powder after lyophilisation (Ishihara *et al*, 2000a). The weight fraction of NAC-Hep (non-reduced IO_4_-LMW-Hep) component in the NAC-HCPS was estimated to be 92% using a carbazole assay (Ishihara *et al*, 2001).

### Anti-coagulant activity

Blood plasma was drawn from a femoral artery of a male New Zealand white rabbit (3 kg, Kitayama Labs Inc., Japan) anaesthetised with an intramuscular injection containing xylazine (12 mg) and ketamin (40 mg). The indicated concentration of native Hep, IO_4_-Hep, IO_4_-LMW-Hep or NAC-HCPS was added to 10 ml of plasma, and the activated partial thromboplastin time (APTT) and prothrombin time (PT) were determined.

### Cell culture

Lewis lung cancer cells (3LL) were obtained from the Cancer Cell Repository (CCR) Institute of Development, Aging and Cancer Tohoku University, Sendai, Japan. B16 melanoma cells (B16) were obtained from the RIKEN Cell Bank, Saitama, Japan. These malignant cells and fibroblasts (human dermal fibroblast, Takara Biochemical Corp. Ohtsu, Japan) were grown in Dulbecco's modified Eagle's medium (DMEM, Life Technologies Oriental Inc., Tokyo, Japan) supplemented with 10% heat-inactivated feotal bovine serum (FBS), antibiotics (100 U ml^−1^ penicillin G and 100 μg ml^−1^ streptomycin) under the atmosphere of 5% CO_2_ in air and 100% relative humidity. Human microvascular endothelial cells (HMVEC, Takara Biochemical Corp., Ohtsu, Japan) were grown in medium 199 (Life Technologies Oriental Inc., Tokyo, Japan) supplemented with 10% heat-inactivated FBS, antibiotics (100 U ml^−1^ penicillin G and 100 μg ml^−1^ streptomycin) and 10 ng ml^−1^ fibroblast growth factor-2 (FGF-2, R&D Systems, Minneapolis, MN, USA). The cells used in this study were between the 4th and 8th passage.

### Cell growth assay *in vitro*

Fibroblasts, 3LL and B16 cells (5×10^3^ per well) were seeded on 96-well tissue culture plates (Falcon) in 100 μl of DMEM containing the same FBS and antibiotics as mentioned above, as well as the indicated concentration of either Hep, IO_4_-Hep, IO_4_-LMW-Hep or NAC-HCPS, and grown for 3 days. Human microvascular endothelial cells (5×10^3^ per well) were seeded on 96-well tissue culture plates in 100 μl of medium 199, containing (i) the same FBS and antibiotics as mentioned above, (ii) the indicated concentration of either Hep, IO_4_-Hep, IO_4_-LMW-Hep or NAC-HCPS, and (iii) one of the growth factors (either 4 ng ml^−1^ of VEGF_165_, 10 ng ml^−1^ of FGF-2 or 20 ng ml^−1^ of HGF), and grown for 5 days. After incubation, the depleted medium was replaced with 100 μl of fresh medium including 10 μl of WST-1 reagent (Cell Counting Kit, Dojindo, Kumamoto, Japan) was added to each well, and the optical density (OD) was read at 450 nm in an Immuno Mini plate reader (Nunc InterMed Japan, Tokyo) after 1 h incubation at 37°C. Results are expressed as percentage, using the mean value of controls (without any heparinoid).

### Cell adhesion onto Matrigel-coated plate

Human microvascular endothelial cells (1×10^5^ cells per well) were plated on a Matrigel (20 μl of 0.5% DMEM solution per well, Collaborative Biomedical Products, Two Oak Park, Bedford, MA, USA) coated 24-well tissue culture plate in 1 ml of medium 199, containing the same FBS, antibiotics and the indicated concentration of either Hep, IO_4_-Hep, IO_4_-LMW-Hep or NAC-HCPS without 10 mg ml^−1^ FGF-2. Similarly B16 and 3LL cells (1×10^5^ cells per well) were plated on the Matrigel-coated 24-well tissue culture plate in DMEM, containing the same FBS, antibiotics and the indicated concentration of either Hep, IO_4_-Hep, IO_4_-LMW-Hep or NAC-HCPS. After 1 h incubation, the used medium was removed and the Matrigel-coated wells were gently rinsed five times with PBS to remove non-binding cells. The fresh medium (450 μl) and 50 μl of WST-1 reagent (cell counting kit; Dojindo) was added to each well, and the optical density (OD) of the medium was measured at 450 nm in the Immuno Mini plate reader after 1 h incubation at 37°C.

### Tubular formation of HMVECs

Human microvascular endothelial cells (5×10^4^ cells per well) were seeded on a Matrigel- (50 μl of 1% DMEM solution per well) coated 96-well tissue culture plate in 100 μl of medium 199, containing the same FBS, antibiotics without 10 ng ml^−1^ FGF-2, and the indicated concentration of either Hep, IO_4_-Hep, IO_4_-LMW-Hep or NAC-HCPS as given above. After 8 h incubation, the formation of tube-like structures by HMVEC was examined microscopically and photographed at ×100 magnification. These micrographs were scanned with a film scanner and analysed using an image analyser (NIH Image, Ver. 1.60, NIH, Bethesda. MD, USA). The total length of a tube-like structure was expressed as percentage of the mean value and related to control without any heparinoid.

### Tumour cell invasion assay

The effect of NAC-HCPS on the invasion activity of tumour cells was also evaluated using a growth factor-reduced Matrigel invasion chamber (for a 24-well plate, 8 μm pore size, Becton Dickinson Labware, Bedford, MA, USA) according to the method described by Albini *et al* (1987) with some minor modifications. Briefly, fresh media (DMEM, 0.75 ml) containing 0.1wt% BSA, 10wt% FBS as a chemo-attractant, and the indicated concentration of either Hep, IO_4_-Hep, IO_4_-LMW-Hep, or NAC-HCPS were added to the wells of the plate (the lower chamber). The upper chamber had been pre-coated with Matrigel by the manufacturer. Tumour (3LL or B16) cells were suspended in DMEM containing 0.1% BSA and the indicated concentration of either Hep, IO_4_-Hep, IO_4_-LMW-Hep or NAC-HCPS at a cell density of 5×10^4^ cells ml^−1^, and the cell suspensions (0.5 ml) were added into the upper chamber. After 18 h incubation, non-invading cells were gently removed from the upper surface of the membrane by wiping with a cotton swab. The invaded cells to the lower side of the membrane were stained with 0.4% Trypan blue solution and counted through microscopic observation. Invasion rates were calculated according to the following equation:


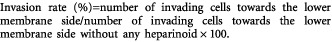


### Tumour growth *in vivo*

Male C57BL/6 mice (6-7 weeks old) were purchased from Clea Japan Inc., Tokyo, Japan. Tumour (3LL or B16) cells were trypsinised and suspended in Hanks' balanced salt solution (HBSS). Tumour cells (1×10^7^) in 100 μl of HBSS were implanted into the dorsal subcutis of the mice. After tumours had reached to a volume of 100∼200 mm^3^ around 14 days (defined as day 1), 200 μl of IO_4_-Hep, IO_4_-LMW-Hep or NAC-HCPS (each 10 mg ml^−1^ of PBS solution) was subcutaneously administered around the tumour daily for another 6 days. Control mice were administered the same volume of only PBS (200 μl per injection). The size of a tumour on day 1 and day 8 was measured with calipers and tumour volume was estimated as length×width× height×π/6. The growth rate of a tumour was then calculated as volume (day 8)/volume (day 1). The 3LL and B16 tumour volumes of control on day 8 were 3200±400 and 4300±500 mm^3^, respectively. Each experimental group was composed of eight mice. Data were compared with the mean volume of the PBS treated group (represented as 100%).

### Vascularity of the tumour

The NAC-HCPS-treated and control tumours of 3LL cells on day 8 were fixed in 10% neutral buffered formaldehyde, and embedded in paraffin for immuno-histochemical study. To evaluate the microvessel density of the tumour, CD34 as an endothelial cell (vessel) marker was stained by an indirect method (Tomisawa *et al*, 1999; Oshika *et al*, 2000). After each section (4 μm thick) was dehydrated and treated with 0.6% hydrogen peroxide in methanol for 45 min, slides were autoclaved for antigen retrieval (121°C 15 min). Slides were then incubated with 5% normal goat serum for 60 min and reacted with rat anti-murine CD34 monoclonal antibody (1 : 20, Hycult Biotechnology, Uden, The Netherlands) at 4°C, overnight. Peroxidase-conjugated anti-rat IgG (1 : 200, Amersham Life Science, Buckinghamshire, UK) was used as the second antibody at room temperature for 60 min and the interactions were visualised with 3,3′-diaminobenzidine-chromogen (DAKO Japan, Kyoto). Finally, nuclear counterstaining was carried out with Mayer's haematoxylin. In each section, five randomised areas (microscopic fields, ×100 magnificant) that were considered to show the largest vessel density, were photographed, and CD34-stained vessels were counted. Each experimental group was composed of six mice. Data have been compared with the average value in PBS treated tumours, defined as 100%.

### Experimental metastasis

Mice were injected intravenously with 3×10^5^ cells of either 3LL or B16 cells in 100 μl PBS through a lateral tail vein. From day 1 to day 7, either IO_4_-Hep, IO_4_-LMW-Hep or HCPS (1 mg per 100 μl PBS) or 100 μl of PBS only was administered intraveneously once a day. All mice were sacrificed day 14 after the tumour cells were injected, and the lungs of each mouse were removed. Lungs were then fixed in Bouin's solution overnight and the surface tumour nodules were counted under a stereoscopic microscope. The numbers of 3LL and B16 tumour nodules in control (PBS-treated) were 250±50 per mouse and 180±30 per mouse, respectively. Each experimental group was composed of eight mice. Data were evaluated against the mean values of the PBS treated group (represented as 100%).

### Statistical analysis

All summarised data are expressed as the mean value±s.e. Comparisons between means of multiple groups were analysed by one-way analysis of variance and Scheffe's multiple comparisons test. All statistical analyses were carried out using the StatView (version 5.0) statistical package (Abacus Concepts Inc., Berkeley, CA, USA). All animal experiments have been carried out with ethical committee approval of the National Defense Medical College, Tokorozawa, Saitama, Japan. The ethical guidelines that were followed meet the standards required by Cancer Research UK guidelines (Workman *et al*, 1998).

## RESULTS

### Anti-coagulant activity of NAC-HCPS

**Figure 2:**
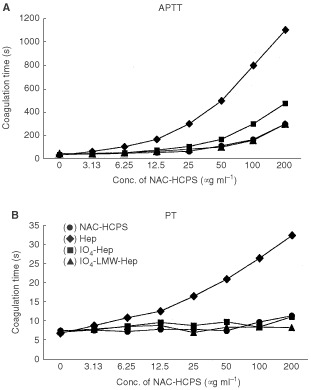
Anticoagulant activity of NAC-HCPS. APTT (**A**) and PT (**B**) of rabbit plasma containing various concentrations of NAC-HCPS, Hep, IO_4_-Hep and IO_4_-LMW-Hep were measured, as has been described in Materials and Methods.

Addition of each Hep, IO_4_-Hep, IO_4_-LMW-Hep, and NAC-HCPS prolonged the coagulation time of rabbit plasma in a dose-dependent manner (Figure 2A

, APTT). APTT of the rabbit plasma without any heparinoids was about 30 s. At a concentration of 100 μg ml^−1^, Hep greatly prolonged APTT to about 800 s, while IO_4_-Hep, IO_4_-LMW-Hep and NAC-HCPS prolonged APTT to about 300, 160 and 160 s, respectively. As shown in Figure 2B, the PT of rabbit plasma without any heparinoid is about 7 s. While Hep strongly prolonged PT to about 27 s at the concentration of 100 μg ml^−1^, IO_4_-Hep, IO_4_-LMW-Hep and NAC-HCPS only prolonged the PT to 8 to 10 s. Since the periodate oxidation of Hep is known to destruct a penta-saccharide structure which interacts with antithrombin III (Conrad and Guo, 1991), the remainder of the anticoagulant activities of IO_4_-Hep, IO_4_-LMW-Hep and NAC-HCPS may result from interactions with other anticoagulant factors, such as heparin co-factor II (Bourin and Lindahl, 1993).

### Effect of NAC-HCPS on cell growth *in vitro*

**Figure 3:**
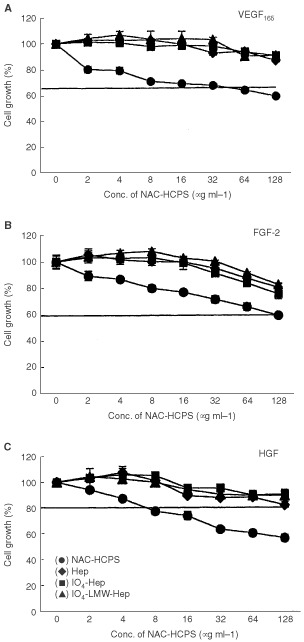
Effect of NAC-HCPS on growth factor-stimulated HMVEC growth. (**A**) VEGF_165_, (**B**) FGF-2, (**C**) HGF. Cell growth incubated with a growth factor in the absence of heparinoid was defined as 100% growth, and the data were calculated as a percentage. The horizontal line in each panel represents the level of cell growth obtained in the absence of both growth factor and heparinoid. The results represent the mean±s.e. in triplicate.

Human microvascular endothelial cells were able to grow in medium in the presence of 10% FBS without addition of a specific growth factor. When VEGF_165_, FGF and HGF were added to the culture medium, the growth rate of HMVEC increased to 1.2–1.7-fold when compared to the control. Horizontal lines in Figure 3A,B

,C show the level of cell growth in DMEM containing 10% FBS in the absence of exogenous growth factors. While addition of low concentrations (below 8 μg ml^−1^) of Hep, IO_4_-Hep or IO_4_-LMW-Hep to the medium did not influence the growth of HMVEC in the presence of each growth factor, high concentrations (more than 32 μg ml^−1^) slightly inhibited the growth in a dose-dependent manner (Figure 3A,B,C). On the other hand, NAC-HCPS inhibited the growth factor-induced HMVEC growth in a dose-dependent manner, even at low concentrations (<2 μg ml^−1^).

**Figure 4:**
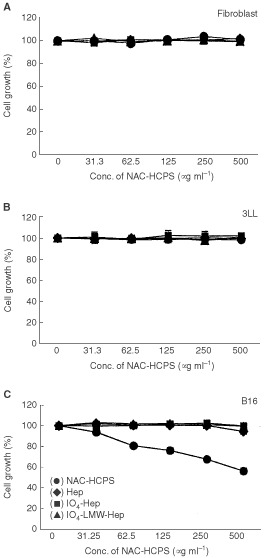
Effect of NAC-HCPS on fibroblast, 3LL and B16 cell growths. The cell growth in the absence of heparinoid was defined as 100% growth, and the data were calculated as a percentage. The results represent the mean±s.e. in triplicate.

The doubling times of fibroblast and 3LL cell growth in DMEM containing 10% FBS and antibiotics were 25 and 17 h, respectively, and the cell growth was not influenced upon addition of either Hep, IO_4_-Hep, IO_4_-LMW-Hep or NAC-HCPS up to 500 μg ml^−1^ (Figure 4A,B

). However, only NAC-HCPS inhibited B16 cell growth in a dose-dependent manner (Figure 4C). The doubling time of B16 cell growth in the control culture and 500 μg ml^−1^ NAC-HCPS containing culture were 18 and 23 h, respectively.

### Effect of NAC-HCPS on tubular formation of HMVECs

**Figure 5:**
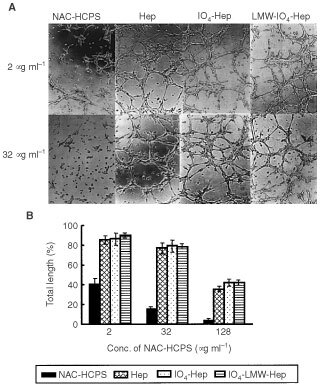
Effect of NAC-HCPS on tubular formation of HMVEC. (**A**) Photomicrographs (original magnification: ×100) of the tubular formation of HMVEC cultured with 2 or 32 μg ml^−1^ of NAC-HCPS, Hep, IO_4_-Hep and IO_4_-LMW-Hep for 8 h on Matrigel-coated plates. Results are representatives of three independent experiments. (**B**) The quantitative evaluations of tubular formation of HMVEC cultured with various concentrations of NAC-HCPS, Hep, IO_4_-Hep and IO_4_-LMW-Hep.

Human microvascular endothelial cells are well known to form capillary-like tubular structures when seeded on Matrigel (Collen *et al*, 2000). The effect of NAC-HCPS in culture media on this tubular formation of HMVEC was compared to that of Hep, IO_4_-Hep and IO_4_-LMW-Hep. Figure 5A

shows representative microphotographs of tubular formation of HMVEC cultured in the presence of respectively 2 or 32 μg ml^−1^ of NAC-HCPS, Hep, IO_4_-Hep and IO_4_-LMW-Hep. While Hep, IO_4_-Hep, and IO_4_-LMW-Hep showed a low inhibitory effect on the tubular formation up to concentrations of 100 μg ml^−1^ (Figure 5B), NAC-HCPS exhibited a strong inhibition on the tubular formation, even at low concentration (2 μg ml^−1^, Figure 5A,B).

### Effect of NAC-HCPS on tumour cell invasion

**Figure 6:**
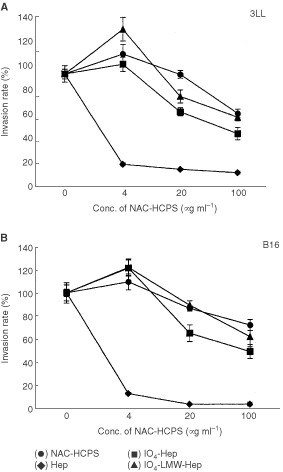
Effect of NAC-HCPS on tumour cell invasion. Tumour cells (3LL (**A**) and B16 (**B**)) were seeded on a 8 μm pore size membrane coated with Matrigel. After 18 h incubation, invaded cells were stained and counted. The invasion rates were calculated as described in Materials and Methods. Results represent the mean±s.e. of four independent determinations.

The inhibitory effect of NAC-HCPS on the migration and invasion of tumour cells (3LL and B16) was examined using a Matrigel invasion chamber (Albini *et al*, 1987). When 3LL and B16 cells were cultured on the Matrigel invasion chamber without any heparinoid, about 200 and 80 cells were invaded into the lower side of the membrane, respectively. While both cell types showed a slightly enhanced invasive activity in the presence of 4 μg ml^−1^ of Hep, IO_4_-Hep, and IO_4_-LMW-Hep, the invasive activities were reduced in the presence of high concentrations (100 μg ml^−1^) of Hep, IO_4_-Hep, and IO_4_-LMW-Hep (Figure 6A,B

). On the other hand, the invasive activity of both cell types in the presence of NAC-HCPS was strongly inhibited, even at a low concentration (4 μg ml^−1^). Therefore, it is suggested that NAC-HCPS possesses an anti-invasive activity for tumour cells.

### Effect of NAC-HCPS on endothelial and tumour cell adhesions to Matrigel-coated plates

**Figure 7:**
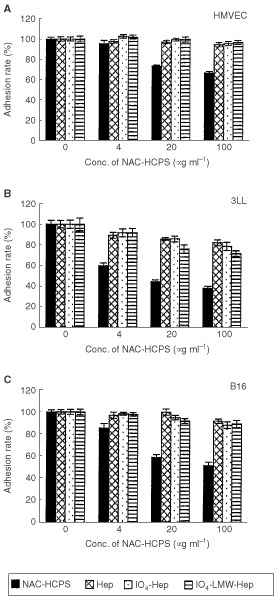
Effect of NAC-HCPS on HMVEC, 3LL and B16 cell adhesions on Matrigel-coated plates. HMVEC, 3LL and B16 cells were plated on Matrigel-coated plates and incubated for 1 h. The bound cells were quantified as described in Materials and Methods. The results represent the mean±s.e. in triplicate.

The adhesions of HMVECs and tumour cells (3LL and B16) to Matrigel-coated plates may be an important first step in the tubular formation of HMVECs on the Matrigel, as well as tumour cell invasion through the Matrigel invasion chamber, respectively. We examined the effect of NAC-HCPS on both HMVEC and tumour cell adhesion to Matrigel-coated plates. All HMVEC, 3LL and B16 cells adhered to the Matrigel-coated plates within 1 h in the absence of NAC-HCPS (Figure 7

) showing spreading shapes on the surface. On the other hand, NAC-HCPS inhibited adhesion of all the HMVEC, 3LL and B16 cells to the Matrigel-coated plates in a concentration-dependent manner (Figure 7). Furthermore, almost all cells of these three cell types retained their spherical shapes in the presence of high concentrations of NAC-HCPS (20 and 100 μg ml^−1^) up to 5 h (data not shown). However, neither Hep, IO_4_-Hep nor IO_4_-LMW-Hep showed the cell shape-retaining effect.

### Effect of NAC-HCPS on tumour growth *in vivo*

**Figure 8:**
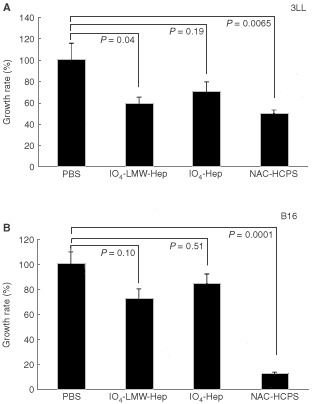
Effect of NAC-HCPS on subcutaneous induced tumour growth of 3LL (**A**) and B16 (**B**) cells in mice. Tumour cells (1×10^7^) were implanted into the dorsal subcutis of mice. After tumours reached a measurable size (100∼200 mm^3^), 2 mg per 200 μl PBS of NAC-HCPS, IO_4_- Hep, IO_4_-LMW-Hep or PBS (200 μl) only was daily administered subcutaneously in the vicinity of the tumour for 7 days. Growth rates were calculated as described in Materials and Methods. Data were compared with the average tumour volume of the PBS treated group, defined as 100%.

A measurable tumour (tumour volume: 100∼200 mm^3^) was formed 14 days after implantation of tumour cells (3LL or B16). As shown in Figure 8A

, IO_4_-Hep and IO_4_-LMW-Hep reduced subcutaneous induced tumour growth of 3LL cells to various extents. NAC-HCPS more strongly inhibited tumour growth to about 40% of the PBS treated group (*P*= 0.0065, *vs* PBS) than IO_4_-Hep and IO_4_-LMW-Hep. On the other hand, while IO_4_-Hep and IO_4_-LMW-Hep did not significantly reduce the subcutaneous induced tumour growth of B16 melanoma (Figure 8B), NAC-HCPS significantly inhibited the tumour growth to about 10% of the PBS treated group (*P*<0.0001, *vs* PBS).

### Effect of NAC-HCPS on vascularity of the tumour *in vivo*

**Figure 9:**
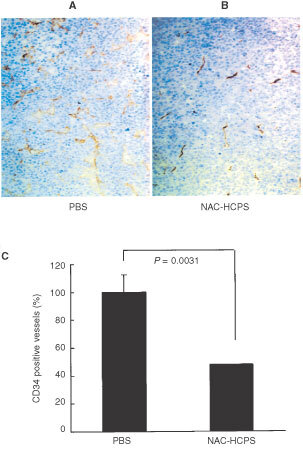
Effect of NAC-HCPS on 3LL-tumour vascularisation. Vascularisation of the 3LL-tumour, evaluated immuno-histochemically with anti-murine CD34, markedly decreased in NAC-HCPS treated 3LL-tumours (**B**) when compared with PBS treated 3LL-tumours (**A**). The quantitative evaluation of the vascularisation (**C**) was carried out as described in Materials and Methods.

To evaluate the effect of NAC-HCPS on anti-angiogenesis, immuno-histochemical staining of murine CD34 of NAC-HCPS treated and control tumours of 3LL cells were carried out (Tomisawa *et al*, 1999; Oshika *et al*, 2000). Representative microphotographs of CD34 immuno-localisation in controls (PBS-treated) and NAC-HCPS treated subcutaneous induced tumours are shown in Figure 9A,B

. In PBS treated mice, many CD34 positive stained vessels were diffusely located and clearly formed tube-like structures in the tumour. On the other hand, CD34 positive stained vessels were nearly absent in the NAC-HCPS treated tumours and tube-like structures were not observed. As shown in Figure 9C, NAC-HCPS significantly reduced the number of CD34 positive vessels (*P*=0.0031), suggesting that NAC-HCPS significantly inhibited angiogenesis in tumours.

### Effect of NAC-HCPS on experimental metastasis

**Figure 10:**
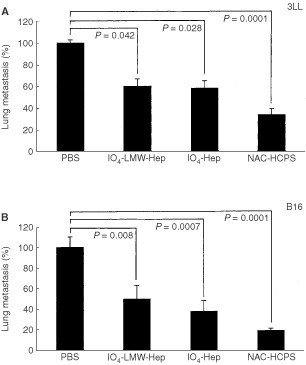
Effect of NAC-HCPS on lung colonisation of 3LL cells (**A**) and B16 cells (**B**) in mice. Both tumour cells (3×10^5^) were intraveneously injected through the lateral tail vein. From day 1 to day 7, either NAC-HCPS, IO_4_-Hep, IO_4_-LMW-Hep (1 mg per 100 μl of PBS) or 100 μl of PBS only was daily administered intraveneously through the lateral tail vein, and colony numbers on the lung surface in each mouse were counted on day 14.

To evaluate the inhibitory effect of NAC-HCPS on lung colony formation of tumour cells (3LL or B16), IO_4_-LMW-Hep, IO_4_-Hep and NAC-HCPS (each 1 mg per 100 μl PBS) was intravenously injected daily for 7 days after injection of the tumour cells. Therefore, Hep was excluded in this study. As shown in Figure 10A

, IO_4_-LMW-Hep, IO_4_-Hep and NAC-HCPS inhibited lung colonisation of 3LL cells. Similarly in Figure 10B, all IO_4_-LMW-Hep, IO_4_-Hep and NAC-HCPS also showed lung colonisation inhibition of B16 cells. Furthermore, in IO_4_-LMW-Hep, IO_4_-Hep and PBS treated mice, a number of 3–20 tumour colonies of 3LL or B16 cells in liver were always observed in each mouse, while in NAC-HCPS treated mice, no colony formation of tumour cells in liver was observed (data not shown). Thus, NAC-Heps, especially NAC-HCPS, possess significant anti-metastasis activity of 3LL and B16 cells.

## DISCUSSION

Heparin is clinically used as an antithrombotic agent, but its high dose use has been limited by its strong intrinsic anti-coagulant property itself, causing severe bleeding complications (Levine *et al*, 1989). If Hep could be modified to minimise its anti-coagulant property and to enhance its activities to inhibit tumour growth and metastasis, then such a modified Hep would be a very useful drug in treating malignant diseases. Periodate-oxidised (IO_4_-) Hep and periodate-oxidised, alkaline-degraded low molecular weight (IO_4_-LMW-) Hep (Fransson and Carlstedt, 1974; Fransson, 1978) are known for not having a specific pentasaccharide structure to interact with antithrombin III (Conrad and Guo, 1991), and therefore its anti-coagulant activity (APTT and PT) is much lower than Hep (Figure 2). We previously reported the preparation of NAC-HCPS using the IO_4_-LMW-Hep (Ishihara *et al*, 2000a). In this study, we have demonstrated that NAC-HCPS inhibits subcutaneously induced tumour growth and metastasis to lung of B16 melanoma and 3LL (Lewis lung cancer) cell line.

In the present study, NAC-HCPS when compared to Hep, IO_4_-Hep and IO_4_-LMW-Hep, has strong anti-angiogenic properties. This inhibitory effect of NAC-HCPS can not be ascribed to cytotoxicity, since it has been found that NAC-HCPS in concentrations up to 500 μg ml^−1^ do not inhibit the HMVEC growth in the absence of those growth factors (data not shown). NAC-HCPS inhibited the adhesion of HMVECs and tumour cells to the Matrigel-coated plate, as well as the tubular formation of HMVEC on Matrigel. The inhibitory effect of NAC-HCPS is probably due to inhibition of the cell adhesion to the Matrigel as well as inhibition of heparin-binding growth factors. Moreover, endothelial cells in the extracellular matrix need binding to adhesive proteins to initiate invasion and migration (McCathy *et al*, 1990). Heparins, especially NAC-HCPS, can effectively bind to various adhesive proteins such as fibronectin, laminin and collagen, and thus may affect cell adhesion and tubular formation. Finally, NAC-HCPS markedly reduced the number of CD34-positive vessels (a marker of microvascular endothelial cells) in subcutaneous 3LL tumours (Tomisawa *et al*, 1999; Oshika *et al*, 2000). The above results demonstrate that NAC-HCPS with its reduced anticoagulant property has a strong anti-angiogenesis. This anti-angiogenesis activity may be the explanation for the observed *in vivo* inhibition of experimental subcutaneous tumour growth.

In this study, it has been demonstrated that NAC-HCPS inhibits the adhesion of tumour cells to Matrigel-coated plates, probably due to the Hep-clustering effect of NAC-HCPS (Figure 7). In addition, immobilization of Hep onto Matrigel was important for the inhibitory effect of NAC-HCPS on tumour cell adhesion to Matrigel. As we reported previously, NAC-HCPS is effectively adsorbed to various polymeric surfaces (Ishihara *et al*, 2000a), collagen (type I)-substratum (Ishihara *et al*, 2001) and Matrigel (data not shown) through a hydrophobic interaction between the hydrophobic surface and polystyrene core of NAC-HCPS. And cell adhesion of tumour cells (B16 and 3LL) were similarly inhibited by immobilisation of NAC-HCPS on the Matrigel (data not shown).

Invasive properties are characteristic of malignant cells, and essential to tumour growth. Tumour cells use specific enzymes to solubilise extracellular matrix during tumour invasion. This degradation of the extracellular matrix takes place at highly localised regions in close vicinity to the cancer, where active proteolytic enzymes outbalance natural protease inhibitors present in the extracellular environment (Basbaum and Werb, 1996). These proteases are produced by either inflammatory cells, stromal cells or the tumour cell themselves (Liotta, 1992). Heparin, chemically modified heparins and related sulphated polysaccharides are known to be effective inhibitors for heparanase (Irimura *et al*, 1986; Vlodavsky *et al*, 1994; Lapierre *et al*, 1996) and various matrix metalloproteases (MMPs) including MMP-1, -2, -3 and -9 (Kenagy *et al*, 1994; Gogly *et al*, 1998). MMP-2 and -9 are suggested to play a major role in metastasis (Kugler, 1999; Westermarck and Kahari, 1999). Heparanase activity has also been found to correlate with the metastatic potential of various types of cancer cells (Nakajima *et al*, 1988). In this study the dose-dependent inhibition of NAC-HCPS on the invasion of tumour cells into Matrigel has been observed (Figure 6). It is possible that an enhanced inhibition of active proteolytic enzymes, as well as inhibition of adhesion of tumour cells to Matrigel by NAC-HCPS result in the strong inhibition of the tumour cell invasions. Tumour cell adhesion to sub-endothelial matrix and the subsequent invasion into the matrix are common pathways for tumour cells to escape from blood flow. The observed inhibition of metastasis by NAC-HCPS seems to be caused through the inhibition of adhesion and invasion of tumour cells (B16 and 3LL).

Our additional studies also have revealed that IO_4_-LMW-Hep and NAC-HCPS have about a 10-fold smaller anticoagulant activity (APTT) than native Hep. The residual anticoagulant activity of NAC-HCPS is probably mediated by interaction with heparin co-factor II, and not antithrombin III (Conrad and Guo, 1991; Lapierre *et al*, 1996). In many tumour types, fibrin is a major component of the initial stroma (Costantini and Zacharski, 1992). Fibrin provides a scaffold for both invasive cancer and endothelial cells, thereby contributing to tumour growth and neo-vascularisation (Dvorak *et al*, 1987). The structure and mechanical properties of the fibrin matrix play a regulating role in the formation of capillary-like tubular structures (Nehls and Herrmann, 1996). Hep with its anticoagulant activity is thus expected to inhibit the formation of fibrin and microthrombi. Whether the low anticoagulant activity of NAC-HCPS contributes to its anti-tumour and anti-angiogenesis properties remains to be determined.

Compared with sulphated polysaccharides like Hep, NAC-HCPS has the advantage of exhibiting less toxicity due to its reduced anti-coagulant activity. The subcutaneous haemorrhages were never observed in mice injected with the same amount of NAC-HCPS, and the NAC-HCPS treated mice lived longer. Furthermore, when 1 mg of NAC-HCPS was intravenously injected daily for 7 consecutive days, only minor increases for the values of GOT (glutamic-oxaloacetic transaminase), GPT (glutamic-pyruvic transaminase), BUN (blood urea nitrogen), and Crea (creatinine) were observed, returning to normal values within 7 days after the final injection (data not shown). However, sufficient data are not yet available of the complete toxicity profile of NAC-HCPS, and standard toxicologic and metabolic studies should be carried out in more detail to confirm the clinical safety of NAC-HCPS.
